# An exploratory spatial analysis to assess the relationship between deprivation, noise and infant mortality: an ecological study

**DOI:** 10.1186/1476-069X-12-109

**Published:** 2013-12-16

**Authors:** Wahida Kihal-Talantikite, Cindy M Padilla, Benoit Lalloue, Christophe Rougier, Jérôme Defrance, Denis Zmirou-Navier, Séverine Deguen

**Affiliations:** 1 EHESP School of Public Health, Rennes, France; 2 INSERM U1085-IRSET, Research Institute of Environmental and Occupational Health, Rennes, France; 3 Lorraine University, Nancy, France; 4 CSTB Scientific and Technical Center for Building, Saint-Martin-d’Hères, France

**Keywords:** Noise exposure, Neighbourhood deprivation, Infant mortality, Spatial analysis

## Abstract

**Background:**

Few studies have explored how noise might contribute to social health inequalities, and even fewer have considered infant mortality or its risk factors as the health event of interest.

In this paper, we investigate the impact of neighbourhood characteristics - both socio-economic status and ambient noise levels - on the spatial distribution of infant mortality in the Lyon metropolitan area, in France.

**Methods:**

All infant deaths (n = 715) occurring between 2000 and 2009 were geocoded at census block level. Each census block was assigned multi-component socio-economic characteristics and Lden levels, which measure exposure to noise. Using a spatial–scan statistic, we examined whether there were significant clusters of high risk of infant mortality according to neighbourhood characteristics.

**Results:**

Our results highlight the fact that infant mortality is non-randomly distributed spatially, with clusters of high risk in the south-east of the Lyon metropolitan area (RR = 1.44; p = 0.09). After adjustments for socio-economic characteristics and noise levels, this cluster disappears or shifts according to in line with different scenarios, suggesting that noise and socio-economic characteristics can partially explain the spatial distribution of infant mortality.

**Conclusion:**

Our findings show that noise does have an impact on the spatial distribution of mortality after adjustments for socio-economic characteristics. A link between noise and infant mortality seems plausible in view of the three hypothetical, non-exclusive, pathways we propose in our conceptual framework: (i) a psychological pathway, (ii) a physiological disruption process and (iii) an unhealthy behaviours pathway. The lack of studies makes it is difficult to compare our findings with others. They require further research for confirmation and interpretation.

## Background

Over the past 20 years, the literature has confirmed that, in developed countries, the leading causes of neonatal morbidity and mortality are related to various adverse pregnancy outcomes such as pre-term birth (PTB) [1-5], congenital malformation [6,7], low birth weight (LBW) [8] and intrauterine growth retardation (IUGR) [8,9]. Moreover, socio-epidemiological research has documented a social gradient in stillbirth and infant mortality, [10-14] despite long term improvements in mortality rates [13-15]. It is well established that both infant mortality and its risk factors are more common among women of low socio-economic status [16,17]. A wide literature describes various deprivation measures related to infant mortality and its determinants as well as to composite indices [16,18-20] or proxy variables of socio-economic characteristics such as income [21-24], education level [21,23,25], unemployment [25,26], occupation category [25], percentage of persons below the poverty level [25] or renting their house [27], percentage of immigrants [22]. In addition to socio-economic and demographic characteristics, environmental factors have been also reported to influence neonatal and infant mortality [28-35].

In order to further explain these health inequalities, researchers on infant mortality and its determinants have advanced the hypothesis that differential environmental exposures might add to role of social determinants [28-35]: “deprived populations are more likely to be exposed to a higher number of environmental nuisances or to a higher level of environmental exposure such as ambient air pollution” [28,31,32,35-37]. In other words, socially disadvantaged inhabitants and ethnic minority populations are more likely to live near traffic or industrial activity than better-off residents. Some authors concluded that the area level effect of air pollution modifies the socio-economic patterns of pre-term birth [28,31,34,35], low birth weight [31], and infant mortality [32,33]. Overall, these studies show that exposure to ambient air particulates yields (i) a three-fold increase in risk of pre-term birth for an increase in PM10 in low-income groups [28], (ii) a significant increase in the risk of all-cause mortality only among infants with low and medium SES [32].

The vast majority of these studies considered air pollution to be the principal environmental nuisance. Few studies have explored how noise might contribute to social health inequalities, and even fewer have considered infant mortality or its risk factors as the health event of interest. Noise exposure is also unevenly distributed across socio-economic groups [38,39] in such a way that exposure to traffic-related noise is particularly high for low socio-economic groups [38] and disadvantaged neighbourhoods [39]. Moreover, noise is increasingly considered to be an important public health problem [40,41]. Residential noise represents a major environmental nuisance affecting a wide population. In the European Union, the World Health Organization (WHO) has estimated that during the daytime, approximately 40% of the population is exposed to residential noise levels in excess of 55 dB (A) while more than 30% is also exposed to the same noise levels at night [41]. Authors have suggested that long-term exposure to excessive noise affects health and well-being through sleep disturbance [42], psychological stress [43], cardiovascular disease [44,45] as well as adverse pregnancy outcomes such as low birth weight [46]. Thus, the consideration of noise as an environmental hazard may improve our understanding of the mechanisms underlying social health inequalities.

In this context, the aim of the present study is to investigate whether spatial clustering of infant mortality exists, as well as whether adjusting for noise exposure and/or socio-economic deprivation can explain any clustering measured at French census block level, in the Lyon metropolitan area. The results of our spatial investigation will be put into the perspective of a theoretical framework of different possible and plausible pathways that may relate adverse pregnancy outcomes to neighbourhood characteristics.

**Figure 1:**
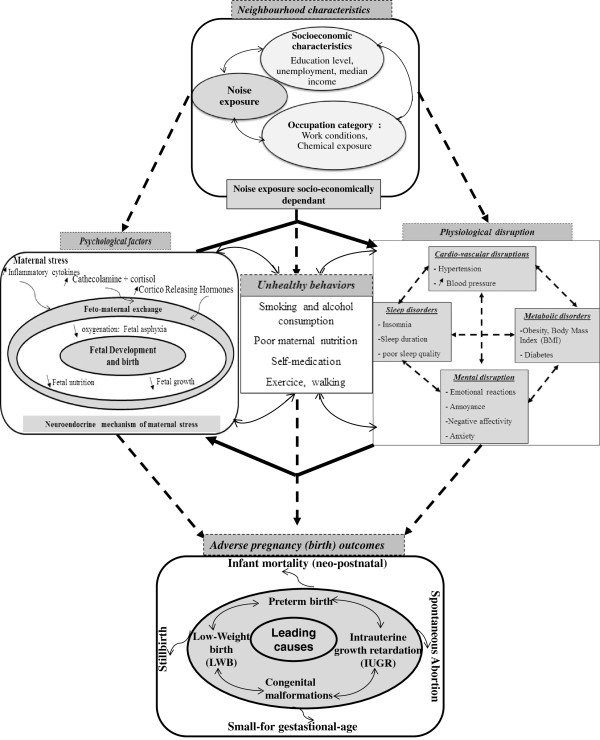
A conceptual model to explain the overlapping relationship between neighborhood characteristics (including exposure to noise and socioeconomic status) and adverse pregnancy outcomes, including neonatal mortality.

Having reviewed the epidemiological and experimental research, we constructed a theoretical model of the underlying mechanisms in which chronic exposure to noise might related to various adverse pregnancy outcomes (Figure 1). This theoretical model highlights three main potential pathways: (i) psychosocial factors as a plausible biologic pathway, based mainly on the role of stress generated by deprived socio-economic status or by exposure to occupational and/or neighbourhood noise; (ii) physiological disruption factors as a hypothetical pathway, based on many physiological disorders related to adverse pregnancy outcomes and generated by the socio-economic characteristics or occupational noise; and (iii) health behavioural disorders related to both socio-economic characteristics and exposure to noise.

The paper is structured as follows. Firstly, we elaborate a theoretical model (Figure 1) that aims to explain the mechanisms through which neighbourhood characteristics may relate to adverse pregnancy outcomes. Secondly, we describe the data and the spatial analysis strategy we used. Finally, our findings are discussed using a theoretical model which we briefly introduce below, and elaborate upon in the discussion section.

Because of limited literature linking infant death to noise, our model includes several adverse pregnancy outcomes, rather than confining itself to infant mortality: pre-term birth, low birth weight and Intrauterine Growth Restriction, which can be viewed as determinants of infant mortality [1,5].

## Methods

### Study setting and statistical units

The setting of our study is the Lyon metropolitan area, an urban area of 515.96 km^2^ located in the Rhône-Alpes region of central-eastern France, with a population of 1,249,216 inhabitants in 1999. Analysis was conducted at French census block level (called IRIS by INSEE, the French National Statistics Institute). Our study area was sub-divided into 510 census blocks/IRIS, each having 2,000 inhabitants on average.

### Health data

**Figure 2:**
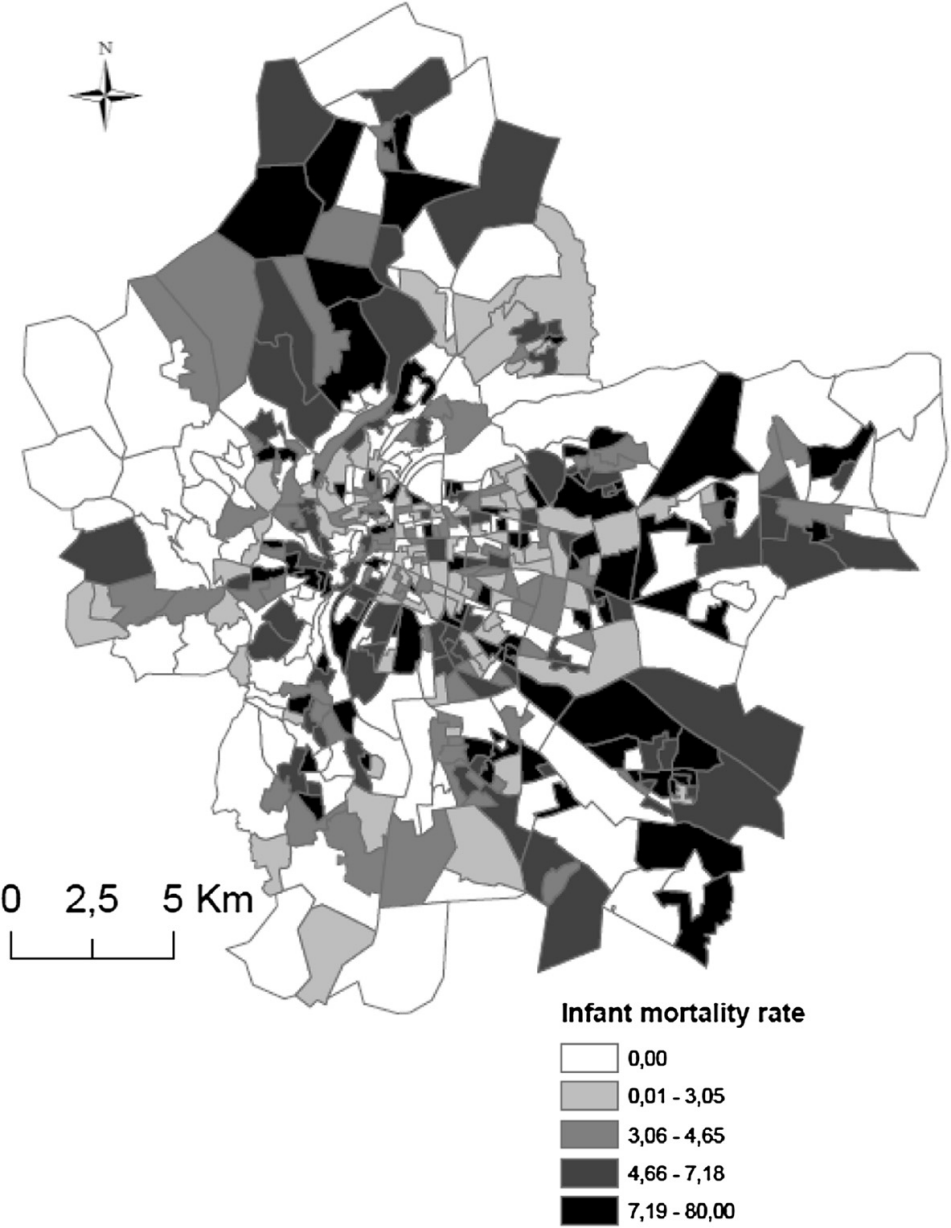
Mapping of spatial display of infant mortality cases across the Lyon metropolitan area.

The dependant variable is infant mortality, defined as all death cases aged <1 year. Data comes from death certificates across all local municipalities. Cases were geo-coded using the parental postal address with the CAZU software from INSEE, which assigns street names and numbers to census blocks. Some cases could not be geo-coded dues to missing. The exhaustiveness of the death data is 96,5%, by comparing the total number of cases collected from the death and birth registries of the study area City Halls with the cases obtained from the National Epidemiological Center for Medical Causes of Death (CepiDc-Inserm). Overall, we collected data on 715 infant death cases in the Lyon metropolitan area between January 2000 and December 2009, including 161 census blocks having 0 deaths. The spatial display of the number of infant deaths per census block is shown in Figure 2. The CNIL (French National Commission for Digitalized Information and Liberty) gave its permission to retrieve geocode and analyze the health data.

### Socio-economic data

Socio-economic and demographic data were obtained from INSEE (1999) at census block level (Table 1). In order to characterize the socio-economic neighbourhood level, we used: i) in a first step, a deprivation index, and ii) in a second step, an array of specific socio-economic variables (including education level, employment and occupation status, and housing characteristics). Principal-component analysis was used to create the deprivation index based on the approach described in Lalloué et al. [47].

**Table 1:** Definition of the socioeconomic characteristics considered as potential predictors of the geographic distribution of infant mortality

**Data**	**Domain**	**Variables**	
**Proxy socio-economic variables**	Occupation	- Proportion of blue-collar workers in the labor force	
		- Proportion of managers in the labor force	
	Education	- People aged 15 years or older with a higher education degree	
		- People aged 15 years or older with at least a lower tertiary education	
		- People aged 15 years or older who did not go beyond an elementary education	
	Immigration status	- Proportion of foreigners in the total population	
	Unemployment	- Proportion of unemployed people in the labor force	
	Housing condition	- Subsidised housing among all primary residences	
	**Characteristics**	**Description**	**Mean [95% CI]**
**Classes of deprivation**	Group 1: low deprivation	Census blocks with high median income, low proportion of households without a car, low proportion with non-owner-occupied primary residences	30935 € [29524–32345]
			9.8% [8.6-11]
			31.6% [28.5-34.8]
	Group 2: moderate deprivation	Census blocks with median income average, medium proportion of households without a car, medium proportion with non-owner-occupied primary residences	23232 € [22627–23838]
			27.4% [25.5-29.4]
			59.2% [56.7-61.7]
	Group 3: high deprivation	Census blocks with low median income, high proportion of households without a car, high proportion with non-owner-occupied primary residences	17072 € [16377–17767]
			33.2% [30.7-35.6]
			78.2% [74.7-81.7]

### Noise exposure data

#### Noise exposure modelling

In 2007, in accordance with requirements from the European Environmental Noise Directive (END, 2002/49/EC) [48], noise nuisances were measured and modelled across the Lyon metropolitan area by the urban Community of Grand Lyon using the GIpSynoise software [49].

The model used follows two stages. First, an acoustic calculation using CadnaA (Computer Aided Noise Abatement), which is a model-based computer program developed by DataKustik (Greifenberg, Germany) [50]; and second, generation of people exposure, using GipSynoise.

The calculation model (CadnaA) incorporates four specific methods recommended by END for calculating current noise levels: (i) NMPB/XPS31-133 (French national computation method – 1996) for road noise; (ii) NMPB –Fer (NF S31-133 :2011 for railway noise [51]; (iii) ISO9613 (Acoustics - Attenuation of sound during propagation outdoors-) for industrial noise; and (iv) ECAC.CEAC DOC.29 (Report on the Standard Method of Computing Noise Contours around Civil Airports) for aircraft noise. These estimations require various input data including information on road, industry, aircraft and railway characteristics, topography, meteorological factors and other various data on the phenomena of sound reflection and diffraction. Using these input data, the acoustic modelling software estimates noise levels with a spatial resolution of 10 × 10 meters at 4 metres above ground level. Based on this information, a noise value corresponding to the most exposed façade is assigned to each building. This value is called ‘building-noise-level’ and, in this work, it is a value including noise coming from all source types ((1) road traffic, (2) aircraft traffic, (3) industries and (4) railway.).

#### Noise exposure calculation at census block level

The metric used to characterize noise in each census block was the European standard Lden indicator (day-evening-night level) - an assessment of daily exposure over a 24 hour period; this indicator takes into account the increased sensitivity of residents to noise during the evening and night by adding an extra factor of 5 and 10 dB (A), respectively, during the corresponding periods [41].

For each census block, we assessed exposure to noise by averaging the Lden estimates of all inhabited buildings within the census block. This approach is derived from the definition of the total noise load of a population L_den,population_ as given in the European Environmental Agency Technical report [52]. The French Scientific and Technical Centre for Building (CSTB) has calculated a mean weighted noise level per census block, defined as:

L¯enerpop,IRISi=10*log1PopIRISI*∑J=1Nbati,jPopbatiji*10LDEN_AL,batji10∀i∈1.....NIRISL⇀ener,pop,IRISi=0ifPopIRISi=0

Pop _Bat__j.i_: population size of residential building in census block/IRIS_i_,

Pop _IRIS__i_: total population of census block/IRIS_i_,

LDEN_AL, bat _j,i_: building-noise level of building j in census block/IRIS_i_,

IRIS i: Only populated census blocks/IRIS

N bat,_i_: Total number of buildings in census block/IRIS i

N _IRIS_: Total number of census blocks/IRIS.

### Analysis

#### Spatial methodology

To evaluate the spatial implications of adjustment for neighbourhood characteristics (noise and deprivation level) on the spatial relocation and provide an explanation of infant mortality risk across Lyon Metropolitan area, the most appropriate approach is spatial method using the spatial scan statistic [53]. We investigated presence, relocation and explanation of spatial distribution of the risk of infant mortality by cluster analysis - the spatial scan statistic implemented in the SaTScan software [54]. This approach, which is used in an increasing number of applications in the field of spatial epidemiology [55], allows exploration of the presence of high risk infant mortality clusters (named ‘most likely clusters’) and their spatial location. The number of cases in each census block is assumed to follow a Poisson distribution.

The procedure works as follows: a circle or windows of variable radius (from zero up to 50% of the population size [56]) is placed at every centroid of the census block and moves across the whole study area to compare the infant mortality rate in the windows with what would be expected under a random distribution. The identification of the most likely clusters is based on a likelihood ratio test [57] with an associated p-value obtained using Monte Carlo replications [58].

#### Analytical strategy and results interpretation

In this approach, the null hypothesis (H_0_) tested is that risk of infant mortality is the same throughout the study area; in other words, the expected infant mortality rate would be randomly distributed in space [54,56]. The alternative hypothesis (H_1_) is that there is an elevated risk of infant mortality within the cluster (one or several geographically close census blocks) in comparison with census blocks outside the cluster.

When the test is statistically significant, this means that the infant mortality rate is not randomly distributed in the Lyon Metropolitan area, i.e. that the identified cluster of census blocks presents a significant increase in infant mortality risk in comparison with census blocks located outside the cluster [59].

The models were adjusted on one or more co-variables, and according to the Kulldorff studies [57], several criteria were used to reject, or not, the H_0_ hypothesis according to the cluster’s localization and statistical significance, and the likelihood ratio value of each model:

 – When, after adjustment, the most likely cluster remains in the same location, (whether or not this location is significant) and its likelihood ratio decreases, the interpretation is that the variable(s) incorporated in the model can partially explain the excess risk [56];

– when the most likely cluster shifts (changes in the location of the centroid of the cluster), this suggests that the covariate(s) in the model can explain the cluster’s excess risk [56]. In addition, another cluster is identified;

– when the most likely cluster disappears totally, it means that the adjusted infant mortality risk is distributed randomly in space.

Thus, spatial analyses were performed in three stages (step by step):

(i) unadjusted analysis, to identify and localize the most likely cluster/s of high risk of mortality

(ii) adjusted analysis for noise exposure **or** socio-economic neighbourhood (deprivation index and the set of socio-economic variables)

(iii)  adjusted analysis for noise **and** socio-economic characteristics at neighbourhood level (including interaction between the two variables)

To incorporate covariates in the model, we classified each census block as having a high, moderate or low level of socio-economic status and noise exposure. When we introduced both noise and socio-economic levels, we also included an interaction term in the model. Because the SaTScan does not allow for an interaction term to be accommodated in the model, we created several dummy variables combining the deprivation and the noise categories.

## Results

### Descriptive results

**Figure 3:**
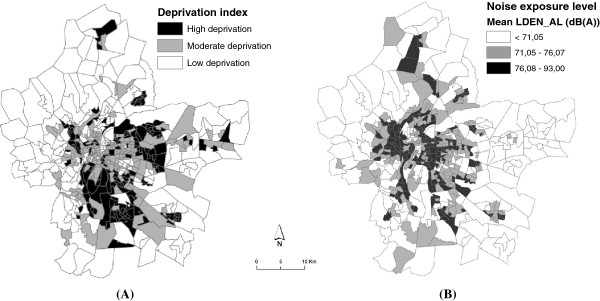
Spatial distribution of the neighborhood socioeconomic index (A) and spatial distribution of neighborhood noise exposure levels modeled across the Lyon metropolitan area (B).

Figure 3A shows spatial distribution of the socio-economic deprivation index at census block level. This map highlights the fact that the wealthiest census blocks are located in the very central and peripheral parts of the study area, while the most deprived blocks are in the central-eastern and southern parts of the metropolitan area.

Figure 3B shows spatial distribution of exposure to neighbourhood noise at census block level in the Lyon metropolitan area. We divided noise levels into tertiles, and observed that census blocks with high noise levels (>71 dB(A)) are concentrated in the central and eastern parts of the metropolitan area, whereas census blocks with the lowest levels (<71 dB(A)) are found in the ring of the city of Lyon. Typically, medium deprivation neighbourhoods had a slightly higher mean noise level. This observation was confirmed by our descriptive analysis, which shows a significant difference of exposure to neighbourhood noise between our 3 deprivation categories (mean noise level = 69.83 dB(A) versus 68. 24 dB(A) and 68.83 dB(A) for medium, low and high deprivation respectively).

#### Spatial results

The statistical results are summarized in Table 2 (unadjusted analysis-stage1), 3 and 4 (adjusted analysis stage 2 and 3 respectively).

**Table 2:** The most likely and secondary clusters resulting from the unadjusted analysis –stage1

	**Confounders**	**Radius (meter)**	**Census block included**	**Expected cases**	**Observed cases**	**RR**^**a**^	**LLr**^**b**^	**P-value**
**Most likely cluster**	None	6966.29	64	116.35	156	*1.44*	*7.52*	*0.09*
**Secondary cluster**	None	0	1	1	7	*3.80*	*4.16*	*0.86*

^**a**^LLr: log likelihood ratio.

^**b**^RR: Relative Risks.

#### Stage 1-Identification of high risk infant mortality clusters

**Figure 4:**
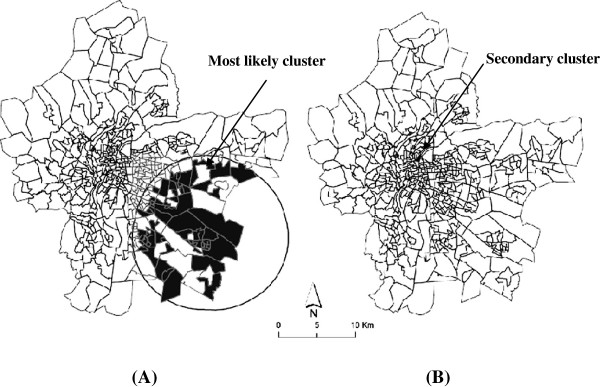
**Mapping of the most likely cluster (A) and secondary cluster (B) of infant mortality.** Legend: Dark area identify census block included in the most likely cluster. This means that the centroid for these blocks falls within the cluster.

Figures 4A and B reveal the location of the most likely cluster and of a small secondary one, respectively. The most likely cluster, in the south-eastern Lyon metropolitan area, has one risk that is 1.44 greater than in the rest of the study area (p-value = 0.09, borderline significant). This cluster comprises 64 census blocks and hosts around 24, 076 inhabitants. The small cluster identified in the immediate northern part of the metropolitan area (RR = 3.8) was not statistically significant (p-value = 0.86) (Table 2).

#### Stage 2 results

Adjusted scan statistical analysis is detailed below according to the variables for which the model was adjusted.

#### Noise exposure and spatial distribution of infant mortality

After adjusting on noise, we found the same most likely cluster (p = 0.10, RR = 7.14), with a slightly lower likelihood value, which decreased from 7.525 to 7.142 (Table 3). We can conclude that noise alone does not explain the excess of infant mortality risk observed in the south-eastern part of the Lyon metropolitan area.

**Table 3:** The most likely clusters resulting from the adjusted analysis –stage2

**Confounders**		**Radius (meter)**	**Censusblock included**	**Expected cases**	**Observed cases**	**RR**^**a**^	**LLr**^**b**^	**P-value**
**Noise exposure**								
**Mean Lden level**		6966.29	64	117.00	156	*1.43*	*7.14*	*0.10*
**Socioeconomic characteristics**								
**Unemployment**		6966.29	64	159.00	156	*1.40*	*6.36*	*0.32*
**Immigration status**		6966.29	64	121.00	156	*1.37*	*5.52*	*0.50*
**Housing conditions**		2795.19	8	13.00	29	*2.25*	*7.17*	*0.10*
**% blue-collar workers**		2795.19	8	15.00	29	*1.91*	*4.77*	*0.60*
**Education**		2795.19	8	15.00	29	*1.94*	*5.02*	*0.70*
**Neighborhood deprivation**		2795.19	8	14.70	29	*1.98*	*5.58*	*0.36*

^**a**^LLr: log likelihood ratio.

^**b**^RR: Relative Risks.

#### Unemployment, immigrant status and spatial distribution of infant mortality

Contrariwise, when adjusted on socio-economic variables, such as unemployment levels (proportion of unemployed people) or immigrant status (proportion of foreigners in the total population), the most likely cluster remained the same size (Figure 4A), with a relatively larger decrease in the likelihood ratio (the value decreases from 7.14 to 6.36 and 5.52 respectively) (Table 3).

The cluster was no longer significant (p = 0.3 and 0.5 respectively), indicating that unemployment and immigration status could explain a relatively large amount of the excess of infant mortality observed in the south-eastern area, in comparison with noise alone.

#### Occupation, housing conditions, education, neighbourhood deprivation level and spatial distribution of infant mortality

Adjusting on housing conditions (characterized by the variable “proportion of subsidised housing among all primary residences”), occupation (% blue-collar workers) or education level (% people over 15 years having a higher-education degree;% people over 15 years with at least a lower tertiary education;% people over 15 years who did not go beyond an elementary education) resulted in the most likely cluster being smaller in size, though located in the same zone (Figure 4A). These clusters where the excess risk of infant mortality span from 1.91 to 2.25 (Table 3, details not shown for all variables) consist of 8 census blocks with about 2,890 inhabitants.

**Figure 5:**
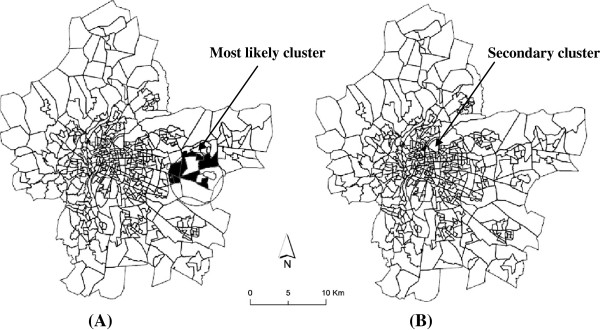
**Spatial shift the most likely cluster (A) and secondary cluster (B) of infant mortality after adjustment.** Legend: Dark area identify census block included in the most likely cluster. This means that the centroid for these blocks falls within the cluster.

The centroid of the cluster shifted (see Figure 5A) and the likelihood ratio decreased from 7.52 to, successively, 7.17, 4.77, 5.58 and 5.02 (Table 3) when the model included housing, occupation, deprivation or the education neighbourhood levels, respectively , indicating that these socio-economic characteristics explain a great part of the excess of infant mortality observed in the unadjusted analysis. The excess risk remaining to be explained becomes not significant (Table 3). The same result was obtained (Figure 5A) when more than one dimension of socio-economic neighbourhood characteristics (such as, for example, housing and education or housing and occupation) was used in the adjusted model (data not shown).

At this stage, occupation, housing conditions, education, and the neighbourhood deprivation level reduce the LLr to a larger degree than unemployment or the immigrant status; these variables explain a relatively large amount of the excess of infant mortality observed in the south-eastern area in comparison with unemployment or immigrant status.

#### Stage 3 results: noise, neighbourhood socio-economic characteristics and spatial distribution of infant mortality

After adjustment for both noise and socio-economic characteristics, in stage 3 of the analysis, the most likely cluster observed in the unadjusted analysis (Figure 4A) now became limited to 8 census blocks (Figure 5A) (with P value from 0.18 to 0.78 and RR from 1.94 to 2.2, see Table 4), yet in the same general location but with the centroid of cluster shifted; also, the likelihood ratio decreased and infant mortality rates did not vary significantly, irrespective of the combination of variables included in the model (Table 4).

**Table 4:** The most likely clusters resulting from adjusted analysis –stage 3

**Confounders**	**Radius (meter)**	**Census block included**	**Expected cases**	**Observed cases**	**RR**^**a**^	**LLr**^**b**^	***p *** **Value**
**Noise and unemployment**	2795.19	8	15.00	29	*1.97*	*5.26*	*0.49*
**Noise and immigration status**	2795.19	8	15.60	29	*1.90*	*4.75*	*0.78*
**Noise and housing conditions**	2795.19	8	13.40	29	*2.21*	*6.92*	*0.18*
**Noise and neighborhood deprivation**	2795.19	8	15.30	29	*1.94*	*5.03*	*0.60*

^**a**^LLr: log likelihood ratio.

^**b**^RR: Relative Risks.

In our study, the significant finding is that while SES had some impact on the LLR of the most likely cluster alone (as shown in Table 3), further adjustment for noise (in Table 4) reduces the LLr to a larger degree than with socio-economic level alone; and this is also larger than the effect of controlling for noise alone.

## Discussion

Our analysis reveals a spatial aggregation of infant mortality located in the south-east of the Lyon metropolitan area. This means that infant mortality rates are not randomly distributed across the study area. After controlling neighbourhood characteristics, the cluster of high risk disappears or shifts according to different scenarios combining socio-economic characteristics and noise neighbourhood levels. Our findings suggest that these factors can partially explain the excess risk of infant mortality. According to our conceptual model (Figure 1), several pathways may explain these associations.

### Noise, socio-economic characteristics and infant mortality

Our study shows that noise contributes to the explanation of spatial distribution of infant mortality beyond SES characteristics, and only after first controlling for SES. This leads us to suspect an effect of chronic exposure to noise. Few studies have explored the relationship between neighbourhood noise and infant mortality – making it difficult to compare our findings with those of others. Our observation is however consistent with previous works which explored occupational and/or aircraft noise and adverse pregnancy outcomes such as perinatal mortality [60], spontaneous abortions [61], pre-term birth [62,63] and low birth weight [64,65] - some of these associations being more pronounced after adjustment on socio-economic condition [64]. These findings were not reported in other papers [66,67]. Noise alone does not seem to be an abortion trigger, yet when combined with occupational characteristics and socio-economic category, Nurminen and Kupra found a more than two-fold excess risk of threatened abortion [68,69].

More recently, both animal and human studies have become more consistent concerning pregnancy outcomes. The Committee on understanding pre-term birth and assuring healthy outcomes [70] provided the theoretical basis for understanding the mechanisms whereby noise could affect pregnancy outcomes. These different pathways were detailed in our conceptual model (see Figure 1), which shows that both neighbourhood socio-economic (described in section above) and noise levels are separately related to adverse pregnancy outcomes by several pathways.

The principal pathway by which noise affects adverse pregnancy outcomes operates through **psychosocial factors** that elicit stress. Human and animal studies demonstrate that subjects exposed to stressors during pregnancy experience a greater risk of spontaneous abortion and of low birth weight [71-74]; also, an increase in stress hormone levels (cathecolamine, cortisol) was found in several studies in workers exposed to noise [74-78]. Such findings were used to explain the results of ecological studies on residents living near airports [46,65,79-81].

A second pathway by which noise could alter pregnancy outcomes and result in pre-term birth [82-84] or foetal growth restriction [85] is **physiological disruption**, a term that refers mainly to sleep disorders such as insomnia, shorter sleep duration and poor sleep quality. Laboratory experiments and epidemiology surveys were two major approaches employed to assess the effect of noise on sleep (see reviews [42,86,87]). Overall, these studies report that sleep disturbance is significantly more frequent in urban populations exposed to traffic noise above 65 Leq dB (A) [88,89] and among populations living near airports [90]. The prevalence of insomnia was found to be higher among inhabitants living closest to busy highways [91]. While experimental studies suggest a hypothetical physiological mechanism for the effect of noise on sleep (see review by Perron et al., 2012) [42], the mechanism underlying the association between sleep and adverse pregnancy outcomes remains unclear. Okun et al. in 2009 proposed a conceptual model involving oxidative stress, endothelial dysfunction and inflammation [92].

Furthermore, the relevant finding emerging from our conceptual model shows an overlap of pathways between neighbourhood characteristics. These suggest that noise effects may add to socio-economic factors on adverse pregnancy outcomes according to two main hypotheses (described below): **psychological factors as a biologic plausible pathway** and **physiological disruption as a hypothetical pathway**.

### Psychological factors as a plausible biologic pathway

The role played by stress generated by deprived socio-economic status [93] or occupational and/or neighbourhood noise [46] is well documented. However, despite substantial literature linking **psychological factors** to adverse birth outcomes, little research has examined potential biological mechanisms to explain these associations [94-96]. Through neuroendocrine and immune mechanisms, the pathway proposed in our model is that chronic stress triggers a series of biological events, through activation of the central autonomic nervous system [97]. As shown in the first part of our model, maternal stress has been implicated in the production of catecholamine, cortisol [46] and inflammatory cytokines [74] in which were found to be increased in both mother and foetus. The release of catecholamine may alter foeto-maternal exchanges by increasing uterine contractions, blood pressure, vasoconstriction of placental vessels and reducing uterine blood flow [46,68,98,99]. In turn, limited foeto-maternal exchanges may affect foetal nutrition and/or oxygenation, and subsequently foetal growth. Therefore, exposure to noise may result in foetal asphyxia [72,100] and elicit both pre-term birth [101,102] and foetal growth restriction [96].

More recent research suggests that the Cortico-Releasing Hormone (CRH) stimulates prostaglandin and oxytocin, the mediators of uterine contraction; therefore it can cause pre-term labour [96,103]. Yet, overall, the biologic pathway underlying stress-induced adverse pregnancy outcomes remains poorly understood.

### Physiological disruptions as a hypothetical pathway

The second part of our theoretical model posits that socio-economic characteristics may be related to many physiological disorders such as cardiovascular [104], metabolic [105,106] and mental disruption [105,107] which are interlinked [108-110], and in turn related to adverse outcomes [111-113]. In this framework, we suggest that annoyance caused by neighbourhood or occupational noise may induce or enhance such disorders specifically cardiovascular [44,45], sleep [42,86] and mental [114,115]. Therefore, as described in the last part of our model, two pathways link these physiological disorders stemming from socio-economic deprivation and/or exposure to noise, infant mortality and its determinants, such as low birth weight and pre-term birth.

Some authors suggest that each of these **physiological disruptions** – such as cardiovascular conditions [111], obesity [112,116], insomnia [117], and mental disorders [113] is directly related to adverse pregnancy outcomes. As described in our model, **unhealthy behaviours** relating to socio-economic characteristics, noise exposure and the physiological disorders mentioned above may affect adverse birth.

### Social inequalities regarding infant mortality

The relationship seen between the neighbourhood socio-economic level and infant mortality confirms previous works regarding occupation [14], unemployment [118], education [14] or neighbourhood deprivation level [16], and pregnancy outcomes (infant mortality, stillbirth, low birth weight or pre-term birth) [17]. Advanced educational achievement enhances acquisition of - and compliance with - healthy practices and recourse to health services [119,120]. Besides educational achievement, other socio-economic characteristics have been related to infant mortality and various adverse pregnancy outcomes via a range of pathways (see conceptual model in Figure 1). Below, we emphasize three specific pathways. **Psychological factors** such as neighbourhood safety [121], stressful life events [93] or lack of social support, cohesive social networks and reciprocal exchanges between residents [122], may impact birth outcomes. Numerous studies have shown that chronic stressors are embedded within and accrued from the environment of women of low socio-economic status [118,123] and/or living in deprived neighbourhoods, who experience more stressful life events during pregnancy than other women [124].

**Occupational conditions** may also influence infant mortality and their determinants [125] through physical fatigue, work duration and intensity, and anxiety or stress which are often associated with such conditions [126].

**Unhealthy behaviours** such as smoking [127] or poor maternal nutrition [128], especially around the time of conception, are known to increase the risk of adverse birth outcomes. Further, some studies suggest that, as a consequence of unequal spatial distribution of various services, [129] pregnant women living in poor or deprived neighbourhoods may have fewer choices and less access to healthy food than their higher-income counterparts [130-132].

Our approach, which uses ecological data, has several limitations. One is the non-inclusion of gender in the analysis, which is known to be a risk factor for infant mortality [133]. Also, for statistical power considerations, we included all mortality cases of infants less than 1 year in our analysis. Focusing on perinatal or neonatal mortality in further studies on larger populations is recommended, and might produce clearer results. Because baby girls have a lower risk of infant and neonatal mortality [134,135], and because the birth gender ratio is uneven across the area census blocks spatial confounding by sex may have partially masked the significance of location of the most likely cluster in analysis.

A second limitation is the absence of individual data such as maternal age, marital status, previous births, parental occupation, and smoking habits. However, in our study we chose a fine geographical resolution scale (IRIS) that is designed by the national statistical institute to be as homogeneous as possible in terms of population size and socio-economic characteristics. The homogeneity of the census blocks ensures minimization of ecological bias, and results stemming from this spatial level lend themselves to analyses that are considered to be close to what can be observed at individual level [136,137]. In other words, the finer the geographical unit, the more homogeneous the population features tend to be, so that the analysis approximates residents’ individual characteristics and environmental exposure patterns. In spite of this, some degree of misclassification inevitably exists in terms of both individual characteristics and environmental exposures, and these results in associations being biased towards the null.

The third limitation concerns the noise data used in the analysis. The modelling technique calls for information on topography, land use, road traffic, population, etc. that may be collected at different periods, leading to uncertainty in matching all the data. Another source of uncertainty is the population calculation for each residential building (Pop _Bat__j.i_) which is computed in proportion to the volume of each building within each census block. For instance, each inhabited building with a height lower than 4 metres is considered to be 4 metres high, according to the methodology used to predict noise levels using the GIpSynoise software [138]. However, compared to crude average, our noise exposure indicator was built to obtain a noise value weighted by the resident population in each building for each census block. It allows the accommodation of census blocks in which a few people are exposed to high noise levels, and larger groups to lower noise levels.

Also, the absence of noise data in the Lyon metropolitan area for each year forced us to use one measure over the 9 years of our study period. No other noise data is available prior to the implementation of the European Environmental Noise Directive. The only available data, therefore, concerns the year 2009. However, according to a report from the Lyon acoustic observatory about the noise evolution between 2008 and 2011, nuisance levels have varied from 0 to 2 dB(A) at most, over this period (i.e. a variation of 2.97% of the exposure level, on average) [139]. Therefore, although we cannot evaluate how noise changes might have influenced our results, our hypothesis is that the variation described by the acoustic observatory is unlikely to alter the contrasts between spatial units and thus would not significantly modify our results. Further, the exposure computation procedure assigns to all residents of a building the value of the loudest face. This tends to over-estimate average exposure levels, but we see no reason why this misclassification should be differential across census blocks. The effect of such random misclassification is to bias the measures of association towards the null. A final limitation of our work is the absence of consideration of air pollution in the analysis. The relationship between noise and the spatial display of infant mortality suggested by our results remains disputable, since noise may be a proxy for other environmental nuisances associated with traffic or industry. Now noise and some air pollutants are strongly correlated. We explored the impact of air pollution in another study [140] conducted in the Lyon metropolitan area, whose results suggest that the socio-economic status and exposure to NO_2_ partially explain the spatial pattern. This means that spatial variation may be due to insufficient adjustment for other risk factors not taken into account in the model, which might explain this remaining heterogeneity in the distribution of infant mortality. In the present paper, we do not take account of noise and air pollution in the same model. They are correlated, with Pearson’s correlation R = 0.57. If we were to use noise exposure and an NO2 together in the same model, we would be faced with collinearity, problems in controlling confounding and the possibility of over- or under-fitting of the nuisance environmental variables. To our knowledge, there is no way that this collinearity can be accommodated in the model.

To our knowledge, such a work had never been performed to explore the effects of neighbourhood noise on the risk of infant mortality. Most related studies examined other adverse pregnancy outcomes (pre-term birth, low birth weight). The **spatial analysis** we chose in order to explore this relationship has been described in only few papers [53,55]. Further, the **conceptual framework** we present in this paper attempts to integrate the many theoretical pathways and hypotheses that are discussed in the literature to relate neighbourhood characteristics to adverse birth outcomes.

## Conclusion

Our findings suggest that there is an association between noise levels, the neighbourhood socio-economic profile and the spatial distribution of infant mortality. However, the relationship between noise and infant mortality is complex and the association we found requires further research for confirmation and interpretation. The conceptual model exposed in the discussion section offers opportunities for new investigations on a topic that has been little explored to date.

## Abbreviations

PM: Particulate matter; CO: Carbon monoxide; NO2: Nitrogen dioxide; PCB: Polychlorinated biphenyls; IRIS: Ilots Regroupés pour l’Information Statistique; INSEE: the French National Statistics Institute; dB: Lden, day-evening-night level; PTB: Pre-term birth; LBW: Low birth weight; IUGR: Intrauterine growth retardation; LLr: Log likelihood ratio; RR: Relative risks.

## Competing interests

The authors declare that they have no competing interests.

## Authors’ contributions

WK performed the spatial analysis, produced the map, conceptual model, drafted the article, and conducted the literature review. C.MP has collected health data, geocoded the cases to IRIS level, and contributed to interpretation of results. BL implemented the statistical models, provided methodological rigor and contributed to the drafting of article and its finalisation. CR and JD have implemented models to assess noise exposure, provided methodological rigor and contributed to the drafting of the article, and its finalisation. DZN, Head of the Environmental and Occupational Health Department at the EHESP and co-principal investigator of the Equit’Area Project, was responsible for quality assurance and rigor in the data analysis, reviewed the drafts of the article and contributed to finalize it. SD, principal investigator of the Equit’Area project studying the role of environmental exposure on health inequalities, monitored the general work, helped with the analysis and interpretation of the results and contributed to draft and finalized the paper. All authors read and approved the final manuscript.
